# A Treatise on the Diseases of Children, with Directions for the Management of Infants from the Birth; Especially Such as Are Brought up by Hand

**Published:** 1785

**Authors:** 


V. *A Treatiſe on the Diſeaſes of Children, with Directions for the Management of Infants from the Birth; eſpecially ſuch as are brought up by hand. By* Michael Underwood, *M. D. Licentiate in Midwifery of the Royal College of Phyſicians in London, and Practitioner at the Britiſh Lying-in Hoſpital.* 12mo. Matthews, *London,* 1784. 288 pages.

THE deſign of this eſſay is to offer a ſuccinct account of the diſeaſes of children, not only to medical readers, but likewiſe to parents and nurſes. The obſervations it contains are thoſe of a judicious and intelligent practitioner; but from what we have ſaid of the plan of the work, we ſhall hardly be expected to enter into a particular account of its contents. Our principal inducement for mentioning it in this place is, for the ſake of introducing to our readers the following deſcription of an anomalous ſpecies of inflammation peculiar to infants,

 and and which ſeems to be new. We ſhall give it in the words of the author :

" Infants are liable to a very dangerous kind

" of eryſipelatous inflammation, not noticed,

" that I know of, by any writer, and which I

" have not often met with but in lying-in hoſ-

" pitals. It never appears, I think, later than

" the month, but moſt frequently ſhows itſelf

" a few days after birth. It attacks the moſt

" robuſt as well as delicate children, and in an

" inſtantaneous manner; the progreſs is rapid;

" the ſkin turns of a purpliſh hue, and ſoon

" becomes exceedingly hard.

" The milder ſpecies of it appears often on

" the fingers and hands, or the feet and ancles,

" and ſometimes upon or near the joints, form-

" ing matter in a very ſhort time. The more

" violent kind is almoſt always ſeated about

" the pubis, and extends upwards to the belly,

" and down the thighs and legs, though I have

" two or three times ſeen it begin in the neck.

" The ſwelling is but moderate, but, after

" becoming hard, the parts turn purple, livid,

" and very often ſphacelate, eſpecially in boys,

" when it falls on the ſcrotum. The penis

" ſwells and puts on that kind of emphyſema-

" tous

" tous appearance which it has in children

" when a ſtone is ſticking in the urethra.

" Various means were made uſe of at the

" Britiſh Lying-in Hoſpital, without ſucceſs;

" though for a time ſome benefit was received

" from ſaturnine fomentations and poultices,

" applied on the very firſt appearance of the

" inflammation, but it ſoon ſpread, and a gan-

" grene preſently came on; or where matter

" had been formed, the tender infant ſunk un-

" der the diſcharge. It is now ſome years

" ſince I propoſed making trial of the bark, to

" which ſometimes a little confectio cardiaca

" has been added, from which time ſeveral

" have recovered. Dr. Garthſhore, one of my

" colleagues, has lately tried the application

" of linen compreſſes wrung out of campho-

" rated ſpirit of wine, in the place of the ve-

" geto-mineral water, which has proved very

" ſucceſsful in ſeveral inſtances; nevertheleſs,

" the greateſt number of infants attacked with

" this diſorder ſtill ſink under its violence, and

" many of them in a very few days."

Dr. Underwood likewiſe deſcribes a peculiar tightneſs and hardneſs of the ſkin over almoſt the whole body, which ſometimes attends that kind of purging in which the ſtools are of a waxy waxy or clay-like confiftence, and ufually takes place in the laft ftage of the difeafe. This complaint, he obferves, was firft noticed by Dr. Denman, who has paid great attention to it. The only child our author has feen recover from this difeafe, took an abforbent aromatic julep.

